# Azithromycin resistance levels and mechanisms in *Escherichia coli*

**DOI:** 10.1038/s41598-019-42423-3

**Published:** 2019-04-15

**Authors:** Cláudia Gomes, Lidia Ruiz-Roldán, Judit Mateu, Theresa J. Ochoa, Joaquim Ruiz

**Affiliations:** 1 ISGlobal, Hospital Clínic, Universitat de Barcelona, Barcelona, Spain; 2 Instituto de Medicina Tropical, Universidad Peruana Cayetano Heredia, Lima, Peru; 3 Center for Infectious Diseases, University of Texas School of Public Health, Houston, Texas USA; 4 Present Address: Molecular Microbiology Area, CIBIR, Logroño, Spain

## Abstract

Despite azithromycin being used in some countries to treat infections caused by Gram-negative pathogens, no resistance breakpoint for *Escherichia coli* exists. The aim of this study was to analyse the levels and mechanisms of azithromycin resistance in *E. coli*. The presence of chromosomal (*rplD, rplV* and *23S rRNA*) mutations, 10 macrolide resistance genes (MRGs) and efflux pump overexpression was determined in 343 *E. coli* isolates. Overall, 89 (25.9%) isolates had MICs ≥ 32 mg/L to azithromycin, decreasing to 42 (12.2%) when assayed in the presence of Phe-Arg-β-Napthylamide, with 35 of these 42 possessing at least one MRG. Efflux pumps played a role in azithromycin resistance affecting the Minimal Inhibitory Concentration (MIC) levels of 91.2% isolates whereas chromosomal alterations seem to have a minimal role. At least one MRG was found in 22.7% of the isolates with *mph*(A) being the most commonly found gene. The *mph*(A) gene plays the main role in the development of azithromycin resistance and 93% of the *mph*(A)-carrying isolates showed a MIC of 32 mg/L. In the absence of a specific resistance breakpoint our results suggest a MIC of 32 mg/L to be considered in order to detect isolates carrying mechanisms able to confer azithromycin resistance.

## Introduction

Infantile diarrhoea is a serious problem in developing countries and remains the second most common cause of death among children under five worldwide. In fact, it causes >800,000 deaths globally per year representing around 10–11% of the annual global child deaths^1,2^. *Escherichia coli* play a relevant role in the death of children by diarrhoea, being involved in more than 120,000 deaths annually of children under 5 years old^1^.

The treatment approach to diarrhoea often does not require the use of antibacterial agents being frequently limited to the replacement of lost liquids and salts by means of Oral Rehydration Salts solutions in order to fight the dehydration risks^3^. However, according to the patient’s nutritional status, the presence of comorbidities, the specific pathogen, illness severity and symptom duration, the use of antimicrobial agents may be required. Ampicillin and cotrimoxazole are the usual first line treatments in most low and middle-income countries^4,5^. Unfortunately, antimicrobial resistance has increased over time, and in different areas these antimicrobial agents are losing their usefulness as a treatment of diarrhoea^4–7^. Since antibiotic resistance is a severe health problem worldwide which can lead to inefficiency of antimicrobial agents and therapeutic failure^8^, surveillance of the development of antimicrobial resistance should be performed, establishing molecular mechanisms of resistance to thereby design alternative treatments.

Azithromycin and other macrolides have been largely used to treat Gram-positive infections and also possess good activity against different Gram-negative microorganisms, such as *Bartonella* spp., *Campylobacter* spp., *Haemophilus influenzae*, or *Neisseria gonorrhoeae*^9,10^. Classically, macrolides present low levels of activity against *Enterobacteriaceae* which have been related to the poor membrane penetration of these antimicrobial agents, preventing their use to treat *Enterobacteriaceae*^9^. Nonetheless, in comparison with other macrolides, azithromycin has a higher basic character^9^. Thus, while low permeability prevents the action of most of macrolide agents against *Enterobacteriaceae*^9^, this basic character confers to azithromycin a true role in the treatment of diarrhoeal infections related to different *Enterobacteriaceae*^11,12^. Thus, azithromycin is a promising alternative because of its excellent activity against most common diarrhoeagenic pathogens such as diarrhoeagenic *E. coli, Shigella* spp., *Salmonella* spp. or *Campylobacter* spp.^9,10^, and has been included in the considered armamentarium to fight against specific *Enterobacteriaceae*^13,14^.

Nonetheless, despite ranking amongst the most frequent etiological causes of diarrhoea^15,16^, and the association of some specific diarrhoeagenic pathotypes with high levels of children mortality^16^, at present no clinical breakpoint for resistance in *E. coli* has been established. However, a Minimal Inhibitory Concentration (MIC) ≥ 32 mg/L or a halo diameter ≤ 12 mm have been proposed as the azithromycin resistance breakpoints in some *Enterobacteriaceae*^17,18^. Furthermore, a series of questions on the use of azithromycin in the treatment of diarrhoeagenic *Enterobacteriaceae* remain to be fully answered. These include questions such as specific azithromycin resistance rates, azithromycin resistance mechanisms in circulation, as well as a more relevant question, such as the effect of different alterations on the final azithromycin MIC.

Chromosomal efflux pumps are bacterial systems involved in the extrusion of molecules from bacteria to the environment, including bacterial products such as siderophores as well as toxics and antibiotics^19^. In this line chromosomal efflux pumps are involved in intrinsic and acquired azithromycin resistance^9,20^. Additionally, target amino acid substitutions in the L4 (*rplD*) and L22 (*rplV*) ribosomal proteins and in *23S rRNA* (*rrlH*) have also been involved in macrolide resistance^9^.

Nonetheless, the most relevant mechanisms of azithromycin resistance in *Enterobacteriaceae* are those encoded in mobile elements^9^. Different Macrolide Resistance Genes (MRGs) have been described, leading to resistance through different pathways such as target modifications produced by rRNA methylases encoded in *erm* genes or macrolides-inactivation, mediated by esterases such as those encoded by *ere(A*) or *ere(B)* genes or by phosphorylases such as those encoded in the *mph*(A) and *mph*(B) genes. Additionally, transferable genes such as *msr*(A), *mef*(A) or *mef*(B*)* have been reported to encode macrolide-efflux pumps^9^.

This study aimed to evaluate the levels and the mechanisms of resistance to azithromycin in a collection of samples of *E. coli* from children with and without diarrhoea. In the absence of a specific azithromycin breakpoint for *E. coli*, we analyse the relationship between specific mechanisms of resistance and MIC levels.

## Results

### Antibiotic susceptibility levels

The MICs of azithromycin ranged between 0.06 mg/L and >256 mg/L, with a MIC_50_ of 8 mg/L and MIC_90_ of 128 mg/L (Table 1).

**Table 1:** Analysis of azithromycin resistance by *E. coli* categories.

	PAβN	Com. (84)	Diarrhoeagenic *E. coli* (259)					
			EPEC (120)	ETEC (41)	EAEC (78)	DAEC (20)	Total DEC	Overall (343)
MIC Range	N	2–>256	0.06–>256	2–256	2–>256	1–>256	0.06–>256	0.06–>256
	Y	0.06–>256	0.06–256	0.25–64	0.5–>256	0.25–128	0.06–>256	0.06–>256
MIC_50_	N	16	8	4	16	16	8	8
	Y	2	1	1	2	4	1	1
MIC_90_	N	128	16	64	>256	128	128	128
	Y	32	2	4	64	32	32	32
R (No./%)	N	23 (27.4)	12 (10.0)	7 (17.1)	38 (48.7)^a^	9 (45.0)^b^	66 (25.5)	89 (36.6)
	Y	13 (15.5)	4 (3.3)	1 (2.4)	21 (26.9)	3 (15.0)	29 (10.8)	42 (12.2)
	*P*	0.0897	0.0671	0.0571	**0.0080**	0.0824	**<0.0001**	**<0.0001**

PAβN: Phe-Arg-β-Napthylamide; Com: Commensal, EPEC: Enteropathogenic; ETEC: Enterotoxigenic; EAEC: Enteroaggregative; DAEC: Diffussely Adherent, DEC: Diarrhoeagenic; MIC: Minimal Inhibitory Concentration (expressed in mg/L); R: Resistance (considering MIC ≥ 32 mg/L); N: Without PAβN; Y: With PAβN; *P*. Differences between resistance levels in the absence and presence of PAβN (highlighted in bold the significant differences found).

^a^EAEC isolates were significantly more resistant than commensal (*P*: 0.006), EPEC (*P < *0.0001) and ETEC isolates (*P* = 0.0007).

^b^DAEC isolates were significantly more resistant than EPEC (*P* = 0.0004) and ETEC (*P* = 0.0302).

**Figure 1:**
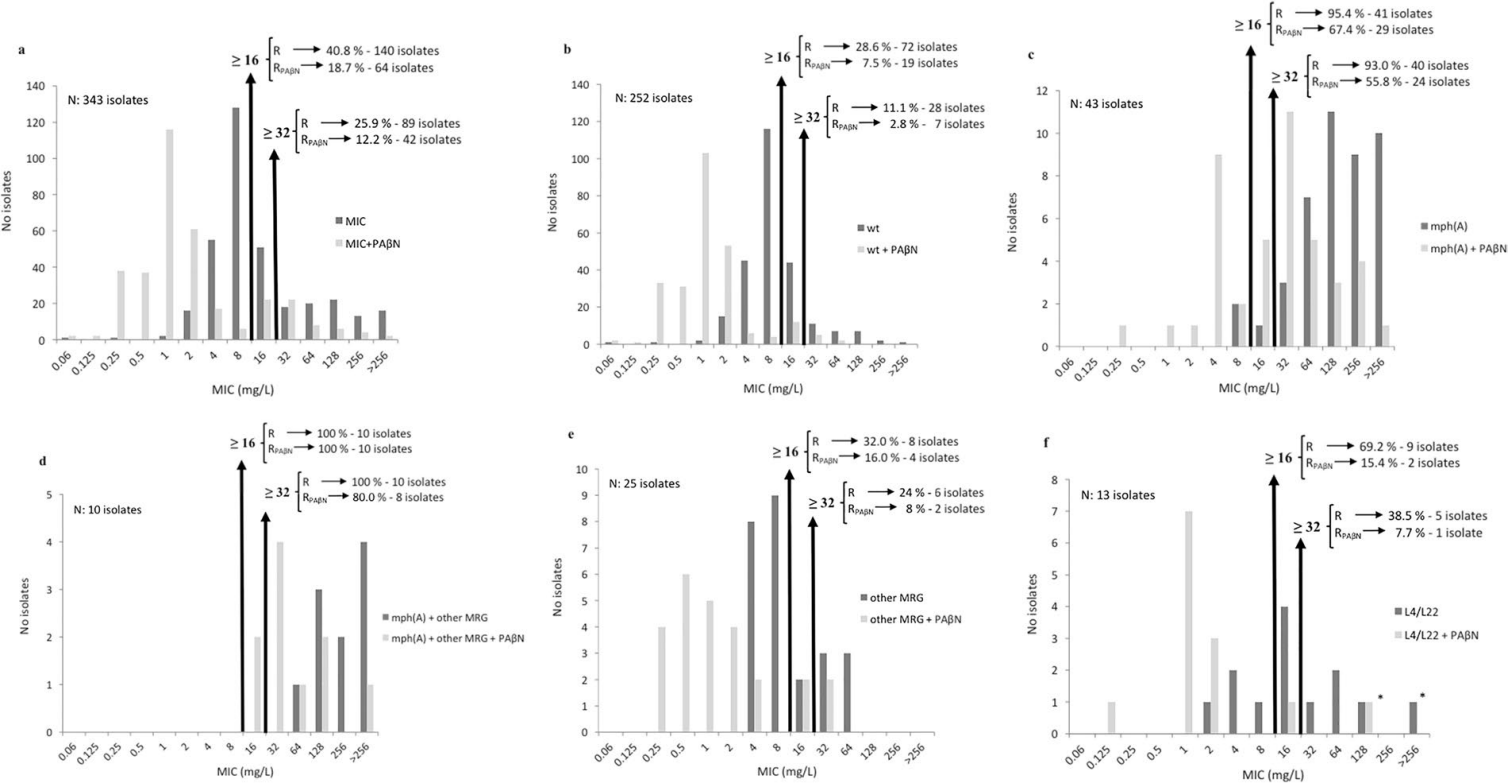
Analysis of Minimal Inhibitory Concentration (MIC) of 16 and 32 mg/L to detect the presence of specific macrolide-resistance mechanisms. R: Resistance; R_PAβN_: resistance in presence of PAβN. (**a**) Overall. (**b**) Isolates in which no sought mechanisms of resistance was found. (**c**) Isolates carrying the *mph*(A) gene alone or with a target mutation. (**d**) Isolates carrying the *mph*(A) gene together other MRG. (**e**) Isolates carrying a MRG different that *mph*(A). (**f**) Isolates carrying only L4 and/or L22 amino acid changes. ^*^The single isolate (isolate 3491) which remains resistant after PAβN addition possesses an unidentified MRG.

**Figure 2:**
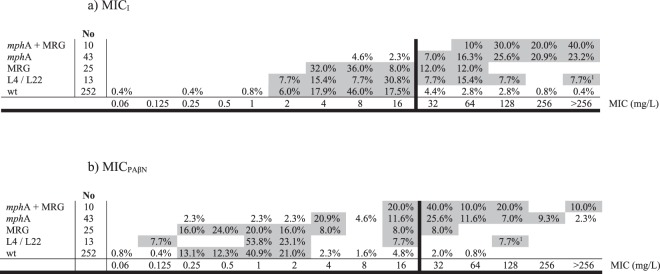
Minimal Inhibitory Concentration (MIC) distribution. MRG: Macrolide resistance gene (other than *mph*(A)); wt: wild type. Any MIC category with ≥5% of the isolates is highlighted in dark grey. If a strain had a L4 and/or L22 mutation(s) and a MRG, then the isolates are included in either the *mph*(A) or MRG category. ^1^One isolate (isolate 3491) in which an unidentified MRG was detected by conjugation.

**Figure 3:**
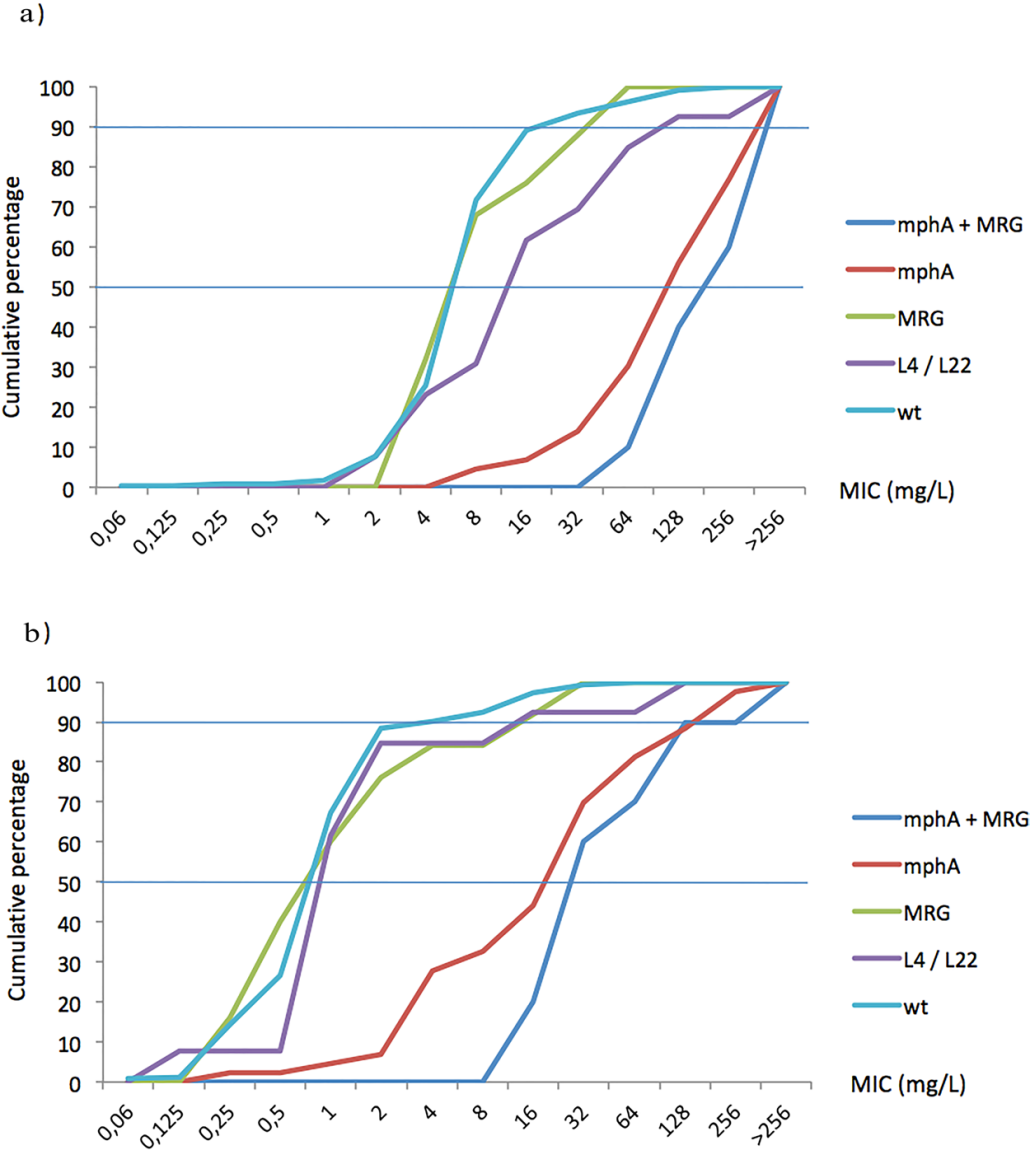
Minimal Inhibitory Concentration (MIC) cumulative curves (**a**) MIC cumulative curve in standard clinical conditions (MIC evaluation in absence of PAβN). (**b**) MIC cumulative curve in presence of 20 mg/L of PAβN. Horizontal lines marks the 50 and 90% of isolates inhibition.

Overall, 140 (40.8%) and 89 isolates (25.9%) had a MIC ≥ 16 and ≥32 mg/L respectively, while only 18.7% and 11.9% (*P* < 0.0001 in both cases) remained with a MIC ≥ 16 and ≥ 32 mg/L respectively when Phe-Arg-β-Napthylamide (PAβN) was added (Table 1, Figs 1, 2 and 3). When the analysis was made comparing diarrhoeagenic and commensal *E. coli* no differences were observed. Nonetheless, when analysing the isolates by pathotypes the levels of resistance of enteroaggregative (EAEC) (48.7%) and diffuse-adhering (DAEC) (45%) were significantly higher than those of enterotoxigenic (ETEC) (17.1%) and enteropathogenic (EPEC) (10%). Moreover, the resistance levels of EAEC isolates were also significantly higher than those of commensal isolates (*P* = 0.0060) (Table 1).

In the presence of PAβN all groups showed decreased levels of resistance, which was significant (*P* = 0.0080) amongst EAEC isolates (Table 1).

### Effect of PAβN

In all cases the isolates were able to grow in the presence of PAβN. As mentioned above the addition of PAβN affected the azithromycin susceptibility levels (Tables 1, 2 and 3, Figs 1, 2 and 3). Overall, when the MIC was established in the presence of PAβN (MIC_PAβN_) the effect of PAβN on the MIC levels was observed in 91.2% of the isolates, independently of the initial MIC (MIC_I_) of azithromycin, with 256 being the maximum MIC_I_/MIC_PAβN_ quotient (from MIC_I_ of 64 mg/L to MIC_PAβN_ of 0.25 mg/L) (Table 2). In 47 out of 89 (52.8%) azithromycin-resistant isolates, the addition of PAβN resulted in a MIC within the range of susceptibility (Table 1, Fig. 1). On the other hand, 35 out of these 47 isolates (74.5%) possessed at least 1 MRG (unidentified in one case - see conjugation results below).

**Table 2:** Analysis of MIC_I_/MIC_PAβN_ quotient.

*E. coli*	MIC_I_/MIC_PAβN_ (N/%)									
	MIC_I/_MIC_PAβN_ ≤ 2		MIC_I/_MIC_PAβN_ > 2							ND*
	0	2	4	8	16	32	64	128	256	
Com (84)		2	24	29	16	7	2	1		3
**DEC (259)**	**5 (1.9)**	**17 (6.6)**	**60 (23.2)**	**103 (39.8)**	**34 (13.1)**	**24 (9.3)**	**2 (0.8)**	**1**		**13 (5.0)**
EPEC (120)	3	2	22	59	14	17	1			2
ETEC (41)		3	9	16	9	2	1		1	
EAEC (78)	2	9	23	23	7	5				9
DAEC (20)		3	6	5	4					2
**Overall (343)**	**5**	**19**	**84**	**132**	**50**	**31**	**4**	**1**	**1**	**16**

MIC_I_/MIC_PAβN_ = 12 (mean effect).

Com: Commensal; DEC: Diarrhoeagenic; EPEC: Enteropathogenic; ETEC: Enterotoxigenic; EAEC: Enteroaggregative; DAEC: Diffussely Adherent; PAβN: Phe-Arg-β-Napthylamide; MIC: Minimal Inhibitory Concentration (expressed in mg/L); MIC_I_: MIC determined in the absence of PAβN; MIC_PAβN_: MIC determined in the presence of PAβN.

ND: MIC_I_ > 256 mg/L. Note that in 10 out of these 16 cases the MIC_PAβN_ was <256 mg/L, and therefore the MIC_I_/MIC_PAβN_ quotient was >2 (e.g.: the quotient >256/128 is at least ≥4), meaning that addition of PAβN affected the final MIC levels. In the remaining 6 cases the possible effect of PAβN was not evaluated because we only were able to determine that the MIC_I_/MIC_PAβN_ quotient was at least 2 (i.e.: the quotient >256/256 is at least ≥2, but not necessarily >2).

**Table 3:** Analysis of azithromycin resistance in the presence and absence of macrolide resistance genes.

	PAβN	Macrolide Resistant Genes (MRGs)			
		Absence (265)	Presence		
			1 MRG (68)		2 MRGs
			*mph*(A) (43)	Other (25)	Overall (10)^a^
MIC Range	N	0.06–>256	8–>256	4–64	64–>256
	Y	0.06–128	0.25–>256	0.25–32	16–>256
MIC_50_	N	8	128	8	256
	Y	1	32	1	32
MIC_90_	N	32	>256	64	>256
	Y	4	256	16	128
R (N/%)	N	81/30.6%	41/95.4%^a,b^	8/32%	10/100%
	Y	21/7.9%	29/67.4%	4/16%	10/100%
	*P*	**<0.0001**	**0.003**	0.3209	1.000

PAβN: Phe-Arg-β-Naphtylamyde; MRG: Macrolide resistance gene; N: Absence of PAβN; Y: Presence of PAβN. MIC: Minimal Inhibitory Concentration (expressed in mg/L). R: Azithromycin resistance (considering MIC ≥ 32 mg/L). *P*: Differences in azithromycin resistance levels related to the absence or presence of PAβN, being significant differences highlighted in bold.

^a^In all cases the *mph*(A) gene was present together with: *erm*(A) − 4 cases; *erm*(B) − 3 cases; *mef*(A) − 2 cases; *ere*(A) − 1 case.

^b^The isolates presenting the *mph*(A) were significantly more resistant than those without MRG or presenting other MRGs (*P* < 0.0001).

Two commensal and 4 diarrhoeagenic isolates presented a MIC_I_ > 256 mg/L and a MIC_PAβN_ ≥ 256 mg/L, thereby not allowing the effect of PAβN to be accurately established.

As a general rule the MIC_I_/MIC_PAβN_ quotient ranged from 4 to 16 (267 isolates, 77.8% of total isolates). The MIC_I_/MIC_PAβN_ mode was 8 (overall, and among commensal and diarrhoeagenic groups), while the mean effect was 12 (Table 2). When the diarrhoeagenic group was subdivided into pathotypes, only DAEC and EAEC showed slight differences (Tables 1 and 2).

Analysing the effect of PAβN in 255 diarrhoeagenic and 82 commensal isolates, a non-significant trend of a higher number of affected commensal isolates was observed (*P* = 0.0810). Thus, the effect of PAβN was not observed in 8.6% and 2.4% diarrheogenic and commensal isolates respectively. Despite the significant effect of PAβN on the MIC of EAEC isolates, 11 (14.7%) were not affected by PAβN. Interestingly, 10 out of these 11 isolates presented MIC_I_ of 64–32 mg/L and MIC_PAβN_ of 32–16 mg/L, with MRGs being detected in only 2 cases. In addition, 3 DAEC isolates (15%) were also not affected by PAβN presenting borderline significant differences with commensal isolates.

### Target mutations

Only 17 out of 263 isolates analysed (6.5%) presented mutations in the *rplD* or *rplV* genes. Thus, 6 isolates had mutations in the *rplD* gene and 7 in the *rplV* gene, while 4 isolates presented amino acid codon alterations concomitantly in both genes. Thirteen of these had a MIC_I_ ≥ 32 mg/L (including 3 presenting mutations in both of the targets analysed), but only one (isolate 3491), in which an unidentified MRG was detected by conjugation (see below), remained resistant when the MIC_PAβN_ was established. In 4 cases were detected concomitant MRGs (Table 4). None of the isolates analysed had mutations in the *23S rRNA* gene.

**Table 4:** L4 (*rplD*) and L22 (*rplV*) amino acid substitutions.

*E. coli*	L4	L22	MRG	MIC ± PAβN	
				N	Y
Commensal	V52I	I4L + L6Q + T72A	—	16	0.125
Commensal	A37S + V52L	wt	—	16	1
Commensal	wt	I4L + K6Q + T72A	—	16	1
Commensal	V52I + D91E + T173N	wt	—	16	2
Commensal	wt	S101T + I103L	—	64	2
DAEC	wt	K83N + D94H + K98N	*mph*(A)	64	4
EAEC	wt	V17I	—	2	1
EAEC	A37T + K74T	wt	—	4	1
EAEC	V120I	wt	—	4	1
EAEC	wt	L46Q	*mph*(A)	64	16
EAEC	K123S	I4L + K6Q	—^a^	>256	128
EPEC	A190V	wt	*msr(*A)	8	0.5
EPEC	D154E	wt	—	8	1
EPEC	V52I + T173N	I4L + K6Q + T72A	*mph*(B)	16	4
EPEC	K123S	I4L + K6Q + T72A	—	32	1
ETEC	wt	L46Q	—	64	16
ETEC	wt	L46Q	—	128	2

PAβN: Phe-Arg-β-Naphtylamyde; MRG: Macrolide resistance gene; wt: wild type. N: Absence of PAβN; Y: Presence of PAβN.

^a^A non-identified conjugative mechanism of resistance was detected (isolate 3491).

### Macrolide resistance genes

Seventy-eight isolates (22.7%) possessed at least one MRG (Table 5). The MRG most frequently found was *mph*(A), which was present in 53 isolates (67.9% of isolates possessing MRG) belonging to all the groups analysed. In 43 cases no other MRG was detected, while in the remaining 10 cases *mph*(A) was detected together with the *erm*(A) gene in 4 cases, the *erm*(B) gene in 3 cases and the *mef*(A) and *ere*(A) gene in 2 and 1 cases, respectively. When more than one MRG was identified within the same isolate the *mph*(A) gene was always present.

**Table 5:** Macrolide resistance genes.

*E. coli*	N	Phosphotransferases		Methylases			Esterases	Efflux Pumps				Overall		
												Isolates		Genes
		*mph*(A)	*mph*(B)	*erm*(A)	*erm*(B)	*erm*(C)	*ere*(A)	*mef*(A)	*mef*(B)	*msr*(A)	*msr*(D)	N	%	N
EAEC	78	21	0	5^a^	3^b^	1	3^b^	3^c^	0	0	1	29^d^	39.8	37
EPEC	120	6	1	0	0	2	0	0	1	1	2	13	10.8	13
ETEC	41	2	0	0	0	1	2	0	0	1	0	6	14.6	6
DAEC	20	10	0	0	1^b^	0	0	0	0	0	0	10^e^	50.0	11
DEC	259	39	1	5	4	4	5	3	1	2	3	58	23.2	67
Comm.	84	14	0	1	2^b^	0	2	0	1	1	0	20^f^	23.8	21
**Overall**	**343**	**53**	**1**	**6**	**6**	**4**	**7**	**3**	**2**	**3**	**3**	**78**	**23.3**	**89**

EAEC: Enteroaggregative; EPEC: Enteropathogenic; ETEC: Enterotoxigenic; DAEC: Diffussely Adherent; DEC: Diarrhoeagenic; Com: Commensal.

^a^4 of them concomitantly with *mph(*A); ^b^1 of them concomitantly with *mph(*A); ^c^2 of them concomitantly with *mph(*A).

^d^Overall the EAEC isolates possess more MRGs than EPEC (*P* < 0.0001) and ETEC (*P* = 0.0113).

^e^Overall the DAEC isolates possess more MRGs than EPEC (*P* < 0.0001), ETEC (*P* = 0.0053) and commensal (*P* = 0.0283).

^f^Overall the commensal isolates possess more MRGs than EPEC (*P* = 0.019).

MRG were significantly more frequent among EAEC and DAEC isolates than among the remaining groups analysed, except when EAEC were compared with commensals. In addition, significant differences were also observed in the presence of MRGs among commensal and EPEC isolates (*P* = 0.0195) (Table 5).

The presence of the *mph*(A) gene was correlated with higher MIC levels (Table 3, Figs 1, 2 and 3), while the presence of other MRGs alone seemed to have a lesser effect. In fact, 40 out of 43 isolates presenting the *mph*(A) gene as a single MRG had MICs ≥ 32 mg/L. Interestingly, those isolates presenting the *mph*(A) gene together with another MRG exhibited slightly higher MIC values than those possessing only the *mph*(A) gene (Fig. 1). The effect of PAβN on the 25 isolates carrying any other MRG was significantly higher (*P* < 0.0001) than in those isolates with the *mph*(A) gene. Thus, only 2 *erm*(B), 1 *ere*(A) and 1 *erm*(A) carrying isolates were classified as non-wt when PAβN was added.

### Conjugation assay

Transconjugants with MICs ≥ 32 mg/L were observed in 16 (24.2%) out of 66 isolates analysed. The *mph*(A) gene was transferred in 14 cases and the *erm*(B) gene in 3 cases (2 together with *mph*(A)). Finally, 1 transconjugant was obtained from a parental isolate (strain 125: MIC_I_ > 256 mg/L; MIC_PAβN_ = 128 mg/L, carrying amino acid changes in L4 [K123S] and L22 [I4L, K6Q]) in which no MRG was previously detected.

### Wt/non-wt phenotypes and MIC levels

Overall, 22 out of 78 (28.2%) isolates carrying at least one MRG presented MIC levels < 32 mg/L. Of these, 3 isolates harbouring the *mph*(A) gene alone (7% of isolates carrying the *mph*(A) gene alone; 3.8% of isolates carrying MRG) and 19 carrying MRGs other than *mph*(A) alone (76% of isolates carrying other MRGs; 86.4% of isolates with MIC < 32 carrying any MRG) having a MIC < 32 mg/L. No isolates possessing more than one MRG presented a MIC < 32 mg/L (Figs 1 and 2). The cumulative MIC curves of wt isolates and those presenting a MRG other than *mph*(A) were similar. The cumulative MIC curves of the isolates possessing target mutations, *mph*(A) and *mph*(A) plus other MRG were sequentially displaced towards higher MIC levels. When the cumulative MICs were established in presence of PAβN the results showed that those belonging to wt isolates, and those presenting MRG or L4/L22 amino acid substitutions were close similar, with only a spurious displacement towards high MIC levels of those non-wt, while isolates possessing *mph*(A) and *mph*(A) plus other MRG were sequentially displaced towards higher MIC levels in a clear manner (Fig. 3).

## Discussion

Diarrhoea-related deaths in children remain among the most relevant health challenges worldwide, being of special concern in low- and middle-income countries^1,2^. In these countries, antibiotic therapy when needed may be crucial to achieve a successful outcome^21,22^. However, antibiotic resistance to commonly used antibacterial agents is dramatically increasing requiring new alternatives.

Regarding the feasibility to considered azithromycin as an alternative to treat diarrhoeagenic *E. coli* in the studied areas, the present study showed moderate azithromycin resistance levels highlighting some concerns about its usefulness as treatment in the absence of antibiotic susceptibility data, especially when EAEC or DAEC isolates are present.

In accordance with what has been previously described^20,23^, the relevant role of PAβN-inhibitible efflux pumps in azithromycin resistance has been demonstrated once more. However, differences related to the specific bacteria groups were observed. The presence of a series of EAEC isolates in which no PAβN-effect was observed opens the door to different options, including the presence of alterations in the outer membrane composition which results in a possible azithromycin impaired permeability leading to an increase in the basal azithromycin resistance levels, combined with lesser efflux pump activity, at least in regard to PAβN-inhibitible efflux pumps. Another possibility is the presence of different patterns of overexpressed efflux pumps. In this line, selecting azithromycin resistant mutants in the presence of PAβN a similar scenario was observed (MIC of 32–16 mg/L with no further PAβN effect). In all these mutants the presence of an overexpressed OmpW was observed^24^. In fact, OmpW has been associated with EmrE, an efflux pump belonging to the small multidrug resistance (SMR) family^9,25^. Furthermore, the overexpression of EmrE has been related to *E. coli* grown in the presence of erythromycin^26^.

In agreement with the presence of up to 7 gene copies and the subsequent need for multiple mutated alleles to visualize an effect on macrolide resistance^9^, in the present study no mutations in the *23S rRNA* gene were observed in the 66 isolates analysed. Regarding L4 and L22, the alterations detected seem to have a minor role in the development of azithromycin resistance, and most might be gene polymorphisms without antibiotic resistance relevance. Regarding the alterations at L4 and L22 observed, to our knowledge only the alterations at amino acid codon K82, D94 and K98 of L22 have previously been described in *in vitro* obtained *E. coli* macrolide-resistant mutants but always concomitantly with other L22 amino acid alterations^27^. The L22 alteration L46Q was present in 3 cases, all having a MIC ≥ 32 mg/L. Although in one case the addition of PAβN resulted in a MIC of 2 mg/L, and another was concomitantly present with the *mph*(A) gene, a possible slight effect of this alteration on macrolide susceptibility cannot be ruled out.

Regarding MRGs, in our series the relevant role of Mph(A) is undoubtable. This finding is in accordance with what has been previously described in *E. coli* and other *Enterobacteriaceae*^9,28–31^. Those isolates with the *mph*(A) gene presented the highest percentages of azithromycin resistance both in the presence and the absence of PAβN. Nonetheless, relevant differences were observed in the MIC levels among isolates carrying the *mph*(A) gene. Thus, while 2 *mph*(A)-carrying isolates had a MIC_I_ of 8 mg/L which decreased to MIC_PAβN_ of 0.25 and 1 mg/L, another 11 isolates in which no other MRG was detected had a MIC_I_ > 256 mg/L which in no case decreased below the breakpoint considered in the presence of PAβN. This heterogeneity may be observed on analysing together different studies performed either in *E. coli* or other closely related *Enterobacteriaceae*^9,28–30^. Different explanations may be proposed, including differences related to expression levels which may be due to the number of copies of the gene related to its genetic environment (e.g.: plasmids with different sizes and copy numbers), with alterations at the promotor sequence or with the presence of other undetected MRGs.

The remaining MRGs, seemed to have a marginal role in azithromycin resistance. In fact, the cumulative MIC curve of these isolates was close to that of wt microorganisms. Nonetheless, those isolates presenting the *mph*(A) together with another MRG ranked among those most resistant and less affected by the addition of PAβN, suggesting a slight contribution of other MRGs to final MIC levels when *mph*(A) gene is present. This finding was also showed when cumulative MICs were established.

Of these MRGs, among *Enterobacteriaceae*, the Msr(A) has only been described in *E. coli* and *Enterobacter* spp.^20,32^. In the present study, the *msr*(A) gene was detected in isolates having MIC_I_ of 8 mg/L, supporting the loss of activity of this gene when cloned in *E. coli*^33^. The other ATP binding transporter studied, Msr(D), it was detected independently of the presence of Mef(A). Moreover, in no case the *mef*(A) and the *msr*(D) genes were detected together. To our knowledge this is the first description of the *msr*(D) gene alone, since it has always been described concomitantly with *mef*(A)^9^. Nevertheless, the presence of polymorphisms in the *mef*(A) primers annealing region cannot be ruled out. While the effect of Msr(D) on the final MIC levels was within the range of those previously described, this dissociation might result in impaired Mef(A)^34^. Contrary to what was observed in the present study, Mef(A) has been described to be frequent in *Enterobacteriaceae*^31^. This difference may be related to the geographical origin of the samples.

This is the first description of Erm(A) in *Enterobacteriaceae*^9,35^. While no data on *erm*(A) functionality in *Enterobacteriaceae* has been found, previous studies have described an impairment in the expression levels of *erm*(C)^36^, which, if combined with a limited gene copy number, might result in a marginal influence on azithromycin MIC levels such as those detected in present study. Regarding Erm(B), the concomitant presence with *mph*(A) detected here in 3 isolates, has also been previously described^30^.

Also Ere(A) had a minimal role in the resistance to azithromycin in the present isolates. This finding is in accordance with the proposed lack of activity of Ere(A) in azithromycin^37^.

There is controversy about the ability of Mph(B) to hydrolyse azithromycin. Thus, while Chesneau and col^38^. have described its inability to confer azithromycin resistance, other authors have established a similar activity on hydrolysing erythromycin and azithromycin^39^. The only isolate of our study that possessed the *mph*(B) gene exhibited an azithromycin MIC of 16 mg/L in the absence of PAβN.

Despite this marginal role of most MRGs in the final azithromycin MIC, the detection of 6 out of 10 MRGs among commensal *E. coli* is noteworthy because of their role as a gene-reservoir^40,41^. Conjugation studies showed that only the *mph*(A) or *erm*(B) genes were transferred alone or together. Additionally, in one case in which no MRG was previously detected, transconjugants were obtained showing the presence of an undetermined MRG. In fact other MRGs have been described in *E. coli*^9,35^. However, it should be noted that the conjugation assay was designed to detect the transference of high levels of azithromycin resistance (>32 mg/L), and thus, if the resistance levels associated with transferable MRGs was lower, the transference of these elements would probably remain undetected.

Although the presence of non-sought mechanisms of azithromycin resistance, similar to observed in the isolate 3491, may not be discharged, and their presence may influences final MIC as observed when *mph*(A) was present concomitantly with other MRG. The fact that the cumulative MIC curves of those isolates presenting target mutations or MRG other than *mph*(A) were only slightly higher than those belonging to wt isolates (on special when role of efflux pumps was discounted with the use of PAβN) confirms the spurious or merely complementary role of these mechanisms as primary azithromycin-resistance cause in *E. coli* and highlight the relevant role of *mph*(A).

Thus, the present data showed that the *mph*(A) gene, is by far, the most effective mechanism of azithromycin resistance present, leading to MIC values higher than 32 mg/L in 93% of the cases, while 88.9% of isolates without mechanisms of resistance remained with MIC levels <32 mg/L. Therefore the use of 32 mg/L seems adequate to suspect the presence of *mph*(A) and in general of non-wt *E. coli* isolates. Nonetheless, the presence of sporadic *E. coli* isolates possessing Mph(A) with MIC values of 8–16 mg/L was also showed. Therefore studies are needed to determine the possible need for more conservative breakpoint.

In summary, the present data demonstrate the presence of azithromycin resistance among intestinal, either pathogenic or not, *E. coli* from the area of Lima, highlighting the need for susceptibility data to adequately use this antimicrobial agent. Moreover, the relevant and hidden role of efflux pumps in the intrinsic levels of azithromycin resistance is highlighted, showing the potential clinical utility of efflux pumps inhibitors. The present data indicate that the majority of isolates harbouring *mph*(A) will have MICs ≥ 32 mg/L. These data, combined with other epidemiological data will be useful to establish an *E. coli* ECOFF value. Clinical data will be needed to establish breakpoints for azithromycin in *E. coli*.

## Materials and Methods

### Bacterial strains

Three hundred forty-three diarrhoeagenic (259 isolates, including 78 EAEC, 41 ETEC, 20 DAEC and 120 EPEC) or commensal (84 isolates) *E. coli* isolates from faeces samples collected in previous studies from children under 5 years of age in periurban areas of Lima (Peru) were recovered from frozen stocks to be included in the study. The *uidA* gene of all grown isolates was amplified as previously described by Walk and colleagues as a quality control^42^.

In all cases the previous studies in which were collected the *E. coli* isolates were approved by the Ethical Committee of the Universidad Peruana Cayetano Heredia, faeces were sampled after informed consent was obtained from parents and/or children legal guardians and all experiments were performed in accordance with relevant guidelines and regulations.

### Antimicrobial susceptibility testing

The MIC of azithromycin was determined by the agar dilution method in accordance with the CLSI guidelines^17^ in the absence (MIC_I_) and presence (MIC_PAβN_) of 20 mg/L of PAβN^20,41^. The effect of 20 mg/L of PAβN on the viability of microorganisms was also assessed. The PAβN effect on the MIC levels was considered when MIC_I_/MIC_PAβN_ > 2. The isolates with a MIC > 256 mg/L that remained unaltered or decreased to 256 mg/L when PAβN was added were not considered in the statistical analysis.

### Ribosomal target gene amplification and DNA sequencing

In a random selected subset of 263 (*rplD* and *rplV* genes) and 66 samples (*23S rRNA*) the presence of point mutations was established by PCR (Table 6), as previously described^23^. The amplified products were recovered with Wizard SV Gel and the PCR Clean Up System (Promega, Madison, Wi) following the manufacturer’s instructions and thereafter sequenced (Macrogen, Seoul, Korea).

**Table 6:** Oligonucleotids used in the study.

Target		Primers		Size (bp)	Ann. (°C)	Ref.
Gene	Prot	Forward (5′ → 3′)	Reverse (5′ → 3′)			
**Macrolide Resistance Genes**						
*ere*(A)	EreA	GCCGGTGCTCATGAACTTGAG	CGACTCTATTCGATCAGAGGC	420	60	^20^
*erm*(A)	ErmA	TCTAAAAAGCATGTAAAAGAAA	CGATACTTTTTGTAGTCCTTC	533	52	^20^
*erm*(B)	ErmB	GAAAAAGTACTCAACCAAATA	AGTAACGGTACTTAAATT	639	45	^20^
erm*(C)*	ErmC	TCAAAACATAATATAGATAAA	GCTAATATTGTTTAAATCGTCAAT	642	45	^20^
*mef*(A)	MefA	AGTATCATTAATCACTAGTGC	TTCTTCTGGTACTAAAAGTGG	345	54	^20^
*mef*(B)	MefB	ATGAACAGAATAAAAAATTG	AAATTATCATCAACCCGGTC	1255	45	^20^
*mph*(A)	MphA	GTGAGGAGGAGCTTCGCGAG	TGCCGCAGGACTCGGAGGTC	403	60	^20^
*mph*(B)	MphB	ATTAAACAAGTAATCGAGATAGC	TTTGCCATCTGCTCATATTCC	868	50	^20^
*msr*(A)	MsrA	GCACTTATTGGGGGTAATGG	GTCTATAAGTGCTCTATCGTG	384	58	^20^
*msr*(D)	MsrD	CCCCAGTTGGACGAAGTAA	TTGTTTTTCCGATTCCATTAC	781	50	^20^
**Macrolide Chromosomal Targets**						
*rplD*	L4	GGCAAGAAAATGGCAGGTCAGATGG	TTCCATCGCAGTAGACGCTTTTTCA	845	56	^23^
*rplV*	L22	CGGTGGAAAGCGGAGACAAGAAGCC	ACCAGTTTTGCGTCCAGTTCAGGCT	925	56	^23^
*rrlH* ^a^	—	TAAGGTAGCGAAATTCCTTGTCG	TGATGCGTCCACTCCGGTC	756	61	^23^
**Other**						
*rep* ^b^	—	GCGCCGICATGCGGCATT	—	MB	40	^23^
*uidA*		CATTACGGCAAAGTGTGGGTCAAT	CCATCAGCACGTTATCGAATCCTT	658	55	^42^

DNA: Amplified gene or DNA fragment; Prot: Encoded protein; Size: Amplified product size; Ann: Annealing temperature; MB: Multiband (having different and no related sizes).

^a^Encode the *23S rRNA*; ^b^Primer designed to amplify the space between Repetitive Extragenic Palindromic (REP) sequences.

### Transferable azithromycin resistance mechanism detection

The presence of 10 established MRGs (*erm*(A), *erm*(B), *erm*(C), *ere*(A), *mph*(A), *mph*(B), *msr*(A), *msr*(D), *mef*(A) and *mef*(B) genes) was sought in all isolates by PCR (Table 6). In all cases negative and positive controls (microorganisms carrying the MRGs included in the study) were used to validate the results. Additionally random selected positive PCRs were sequenced.

### Conjugation assays

A total of 66 isolates with a MIC ≥ 32 mg/L were selected to determine the transferability of the MRGs. The conjugation was carried out in Luria-Bertani broth (Conda, Madrid, Spain) with azide-resistant *E. coli* J53 as a recipient strain. Transconjugants were selected in plates containing 150 mg/L of sodium azide and 32 mg/L of azithromycin. In order to avoid considering possible contaminations the relationship of transconjugants and the respective recipient strain was established by REP-PCR^23^. The amplification of the MRGs present in the donor and derived transconjugant strains was performed by PCR as mentioned previously.

### Statistical analysis

The Fisher exact test was used for statistical analysis. *P* values ≤ 0.05 were considered significant. A microorganism was considered “wt” when no sought mechanism of resistance other than PAβN inhibitable efflux pumps was identified.
